# Ventral Hernia, Six Years Irreducible

**Published:** 1855-10

**Authors:** 


### RECORD OF MEDICAL SCIENCE.

*Ventral Hernia, six years irreducible.—Treatment by Ice.—Reduction.* (Under the care of Mr. Lloyd.)—The reduction of herniæ, which without symptoms have long been irreducible, is not always a matter of moment. They generally consist of omentum, and, excepting they occasion inconvenience by their size, or by dragging upon the other viscera of the abdomen, it is, perhaps, as well to allow them to remain. While in the sac the omentum acts as a sort of plug, and, to a certain extent, lessens the likelihood of the protrusion of bowel. There are cases, however, in which it becomes very desirable to effect reduction, and as a guide to the practice most likely to be successful, the following brief narrative seems worthy of being placed on record.

A few weeks ago Richard B., a laboring man, aged 54, stout and fat, was admitted into Pitcairn ward, on account of an adipose tumor in the right thigh. At the same time he mentioned also a second tumor of much smaller size, and to which he did not attach much importance, which was situated in the abdominal wall. On examination, a lump about the size of an egg, flattened, and giving to the finger exactly the impression of a mass of omentum, was found about an inch to the left of the median line, midway between the umbilicus and pubes. The margins of the aperture in the muscular parietes could be plainly distinguished, and, as the mass received a very decided impulse during coughing, no doubt was felt as to its nature. The man stated that he had known of its existence for six years, during which time he had never been able to get it back. He had never had any symptoms of strangulation, but ever since the tumor had existed, he had been liable at times to sickness and vomiting, especially after eating freely. A sense of dragging was he said, very frequently present, when at work, but it had never prevented him from following his occupation. His most comfortable position was the recumbent.

The fatty tumor having been excised, during the time that the man was confined to bed on account of the wound, Mr. Lloyd several times attempted to reduce the hernia, but without success. On each occasion it would seem to diminish somewhat, but could never be quite returned.

Such being the case, it was decided to try the ice treatment, and a bladder of ice was accordingly ordered to be kept constantly applied over the part. Mr. Jowers, the House-Surgeon, was to employ the taxis perseveringly every morning. Under this plan complete reduction was effected on the ninth day. No restrictions in diet had been enforced, and the application of the ice had not been quite constant. The man was subsequently furnished with a truss, and up to the time of his discharge no reprotrusion had ever taken place.

The case of a young man, under the care of Mr. Hilton, in Guy's Hospital, for whom a large scrotal hernia, which had been six months irreducible, was returned under the ice and abstinence plan, may be found, detailed in our Reports for May 28th, 1853, page 554. In it the necessity for reduction was great, as the man had been quite prevented from doing work. Five day's treatment were required, during which time the man was made to almost abstain from fluids, while diuretics and purgatives were freely given. By these means the quantity of the circulating fluid was reduced as much as compatible with health, and so manifest was their effect, that the tumor which had much decreased in size, and become soft and loose in its sac, ultimately slipped back almost without pressure.

M. Malgaigne, to whom belongs much credit for drawing attention to this mode of practice, has recorded several very instructive examples of its success. In one under his care, a scrotal enterocele, unreduced for several years, was got back by seventeen days' treatment. In a second, a scrotal enteroepiplocele, which had been irreducible for seven years, yielded in six days. In both these a very low diet was enforced.—*Med. Times and Gaz.*

